# Supplementary material for: Content-rich biological network constructed by mining PubMed abstracts
Source: BMC Bioinformatics. 2004 Oct 8;5:147. doi: 10.1186/1471-2105-5-147 (PMC528731; doi:10.1186/1471-2105-5-147)
Supplement: Additional File 2 — The original results of the above study (non-essential files are deleted to keep the file size under the limit set by BMC bioinformatics). [file 1471-2105-5-147-S2.bz2 › chilibotAdditionalFile2/dip20/28ID8999548E91/html/CASP3_IL18.html]

 


 **CASP3** and **IL18** 
  
Found 16 abstracts in PubMed, retrieved 16.  
 

 What does Google say? 
 PDF only 
| .edu only 

---

**Interactive relationship** (e.g. stimulation, inhibition, etc)

**Inhibitory relationship**- Both YadA and Irp  [ **CASP3** ]  1 deficient Yersinia mutants were still attenuated in IL 12 and IL 18  [ **IL18** ]  mice but were pathogenic in TNFRp55 mice.  Ref: 12654794 Infect Immun, 2003
- Additionally, a 3.4 fold increase P< ; 0.001 in the activity of the IL 18  [ **IL18** ]  degrading enzyme, caspase 3  [ **CASP3** ] , was found in cardiac tissue, which may explain the observed reduction of cardiac IL 18  [ **IL18** ]  protein abundance.  Ref: 12829183 Cardiovasc Res, 2003
**Neutral relationship**- Caspase 11 not only activates caspase 1, that is required for the maturation of proinflammatory cytokines such as interleukin IL 1 and IL 18  [ **IL18** ] , but also activates caspase 3  [ **CASP3** ] , leading to cellular apoptosis under pathological conditions.  Ref: 11684090 FEBS Lett, 2001
- This could include an increased participation of caspase 3  [ **CASP3** ]  in the apoptotic pathway, consequent to enhanced activation of the enzyme and to the decreased production of IL 18  [ **IL18** ] , which acts as a competitive caspase 3  [ **CASP3** ]  substrate.  Ref: 11566632 Eur Cytokine Netw< MedlineTA>Eur Cytokine Netw, 2001
- Caspase 3  [ **CASP3** ]  inhibitor also reduced IL 18  [ **IL18** ]  production in response to virus infection.  Ref: 11241276 Eur J Immunol, 2001

**Non-interactive relationship** (e.g. studied together, co-existance, homology, etc.)

- Virus infection induces proteolytic processing of IL 18  [ **IL18** ]  in human macrophages via caspase 1 and caspase 3  [ **CASP3** ]  activation.  Ref: 11241276 Eur J Immunol, 2001
- More extensive expression of Fas, Fas L, perforin, caspase 3  [ **CASP3** ] , and IL 18  [ **IL18** ] , but not IL 2, at the acute stage than at the convalescent stage was observed.  Ref: 12011015 Infect Immun, 2002
- Active forms of CPP32  [ **CASP3** ]  were not detectable in either cell type indicating the absence of a degradative pathway of IL 18  [ **IL18** ] .  Ref: 10101231 Brain Res Mol Brain Res, 1999
- Expression of proteins and cytokines participating in the apoptotic process caspase 1, caspase 3  [ **CASP3** ] , Fas CD95, Fas ligand Fas L, perforin, granzyme A, Bax, WAF 1, Bcl 2, interleukin 2 IL 2, IL 18  [ **IL18** ] , and granulocyte macrophage colony stimulating factor in tissue in the acute and convalescent stages of dysentery was quantified at the single cell level by in situ immunostaining.  Ref: 12011015 Infect Immun, 2002
- To test this hypothesis, we found that interleukin IL 1beta, tumor necrosis factor TNF alpha, lipopolysaccharide LPS, granulocyte macrophage colony stimulating factor GM CSF, and IL 18  [ **IL18** ]  appeared to suppress DNA fragmentation, caspase 9, and caspase 3  [ **CASP3** ]  activation in human monocytes.  Ref: 11804874 Am J Respir Cell Mol Biol, 2002
- Increased expression of caspase 3  [ **CASP3** ]  and IL 18  [ **IL18** ]  in tissues with severe inflammation compared to expression in those with mild inflammation was evident, implying a possible role in the perpetuation of inflammation.  Ref: 12011015 Infect Immun, 2002
- Involvement of caspase 1 and caspase 3  [ **CASP3** ]  in the production and processing of mature human interleukin 18  [ **IL18** ]  in monocytic THP.1 cells.  Ref: 9334240 J Biol Chem, 1997
- These results suggest that GCDCA induces apoptosis of BECs through FasR FasL interaction via an autocrine paracrine effect, IL 18  [ **IL18** ]  is responsible for the expression of FasL in BEC, and the up regulation of caspase 3  [ **CASP3** ]  expression is involved in this model.  Ref: 12697255 Hepatol Res< MedlineTA>Hepatol Res, 1997
